# Effect of Frequent SARS-CoV-2 Testing on Weekly Case Rates in Long-Term Care Facilities, Florida, USA

**DOI:** 10.3201/eid2809.212577

**Published:** 2022-09

**Authors:** Lao-Tzu Allan-Blitz, Belal Aboabdo, Isaac Turner, Jeffrey D. Klausner

**Affiliations:** Brigham and Women’s Hospital Department of Medicine, Boston, Massachusetts, USA (L.-T. Allan-Blitz);; Curative Inc., San Dimas, California, USA (B. Aboabdo, I. Turner);; University of Southern California Keck School of Medicine, Los Angeles, California, USA (J.D. Klausner)

**Keywords:** SARS-CoV-2, COVID-19, severe acute respiratory syndrome 2, coronavirus disease, SARS-CoV-2 testing, long-term care patients, respiratory infections, zoonoses, viruses, Florida, United States

## Abstract

We analyzed 1,292,165 SARS-CoV-2 test results from residents and employees of 361 long-term care facilities in Florida, USA. A 1% increase in testing resulted in a 0.08% reduction in cases 3 weeks after testing began. Increasing SARS-CoV-2 testing frequency is a viable tool for reducing virus transmission in these facilities.

Residents of long-term care facilities (LTCFs) in the United States have suffered a disproportionate number of deaths from SARS-CoV-2 (*1*). Testing frequency and result turnaround times may be more relevant than test sensitivity for infection control (*2*,*3*), information that might be used to guide infection control efforts in congregate living facilities (*4*). Semimonthly testing for SARS-CoV-2 was mandated in Florida, USA, for all employees and residents of skilled nursing, elder care, and assisted living facilities beginning June 7, 2020 (*5*). Comparing data from before and after the mandate took effect, we evaluated the effect of testing frequency on weekly SARS-CoV-2 case rates in a real-world setting.

We analyzed deidentified test results from Florida LTCFs during June 2020–April 2021, aggregated with the Nursing Home Provider Information dataset (*6*), which includes the number of facility beds and staff and average aid hours per resident. We further combined our dataset with Johns Hopkins University SARS-CoV-2 time-series data on rates of hospitalization and death (*7*). For the duration of the study period, only care facility staff were permitted entry to the facilities to limit potential sources of infection.

We used a generalized linear mixed regression model with weekly cases as a negative binomial random count variable to assess how the independent variables affected test positivity. We created a naive model based on frequency of SARS-CoV-2 on the day before the start of semimonthly testing to establish a baseline from which to forecast change. We then regressed weekly positive cases from the date semimonthly testing began (given heterogeneity in compliance, modeled as percentage increase in number of tests above baseline), cases from the preceding week, new cases per 100,000 persons in the county, total tests per occupied bed (a surrogate for compliance with semimonthly testing), number of certified beds in the facility, total nurse staffing hours per resident per day (as a surrogate for quality of care), and whether the date of infection was after January 1, 2021, to control for vaccination effects. We analyzed the date that semimonthly testing began as distinct weeks preceding any change in average weekly SARS-CoV-2 cases to investigate a potential time-delay between onset of testing and any potential reduction in case rate. We log transformed all variables except number of cases in the preceding week. We applied a random effect for each nursing home. We performed all analyses using R statistical software (http://www.r-project.org). Review of deidentified data was deemed exempt from institutional review.

We analyzed 1,292,165 SARS-CoV-2 RNA test results from residents and employees among 361 facilities located across 247 Florida postal codes. The average age (+SD) of the study population was 49 years (+31). The average number of new cases among all LTCFs in Florida was 187.9 per week (+148.7), an estimated 0.7 tests/week/occupied bed (+16.2). The average test turnaround time from laboratory receipt was 17.1 hours (+10.4). The average number of tests completed per week was 31,454 (+10,926).

In multivariable analysis, a 1% increase in testing frequency resulted in a 0.08% reduction in SARS-CoV-2 cases (Table). On the basis of the coefficients from the multivariable model, we predicted that a 10% increase in testing frequency would result in a 1% reduction in the weekly long-term care facility case rate among residents. Assuming generalizability on the basis of similar characteristics between LTCFs in our dataset and those reported by the Nursing Home Provider Information dataset (*6*), that reduction would result in 126 fewer cases per week, or 6,552 fewer cases per year, among LTCF residents across the United States.

**Table:** Estimated change in weekly SARS-CoV-2 cases because of increased testing frequency for residents of long-term care facilities, Florida, USA, June 2020–April 2021

Characteristics	Percent change in weekly cases	p value
Time-point of increased testing*		
One week preceding	0.10 (0.03–0.16)	0.003
Two weeks preceding	–0.02 (–0.09 to 0.04)	0.53
Three weeks preceding	–0.08 (–0.14 to –0.02)	0.01
Four weeks preceding	–0.03 (–0.09 to 0.03)	0.34
Nursing home size and quality		
Certified beds per facility	0.006 (0.004–0.008)	<0.001
Aid hours per resident per facility	–0.07 (–0.17 to 0.03)	0.16
Virus conditions		
Cases among long-term care facilities in preceding week	0.09 (0.09–0.10)	<0.001
New county-level cases per 100,000 persons	0.002 (0.001–0.002)	<0.001
Tested after January 2021†	–0.70 (–0.82 to –0.58)	<0.001

*Based on number of tests per occupied bed. †Used as a surrogate for SARS-CoV-2 vaccination.

Our findings suggest that even a 1% increase in testing might be an effective strategy for combating the SARS-CoV-2 pandemic among LTCF residents, although results likely would not emerge until ≈3 weeks after the increase. In the initial 1–2 weeks after semimonthly testing began, isolation and contact tracing interventions likely had not had sufficient time to substantially reduce viral transmission. Conversely, increased testing frequency >3 weeks before data collection was likely too remote to affect case estimates for a given week. A similar finding has been reported; a SARS-CoV-2 infection outbreak in a 135-bed facility was contained predominantly through serial testing of all residents and staff every 3–5 days (*8*). Routine testing is furthermore necessary to identify presymptomatic and asymptomatic cases, which could account for up to 40% of new infections (*9*) and contribute substantially to transmission. Additional studies should evaluate the cost per case prevented of strategies employing varying frequencies of testing to guide use of testing programs among LTCFs.

Our study was limited by the absence of details on interventions started in response to positive tests or whether test samples were from employees or residents. We also were unable to account for concomitant local or statewide interventions such as mask mandates, resident spacing, or indoor ventilation, which might have confounded the effects of testing frequency. Thus, our findings cannot definitively attribute the reduction in case rates to increased testing frequency. However, the large sample size and number of LTCFs included in the analysis, which controlled for several notable confounders, lend credibility to our findings. We advocate increasing SARS-CoV-2 testing frequency as a viable tool for reducing transmission in LTCFs.
